# Predicting bycatch hotspots for endangered leatherback turtles on longlines in the Pacific Ocean

**DOI:** 10.1098/rspb.2013.2559

**Published:** 2014-02-22

**Authors:** John H. Roe, Stephen J. Morreale, Frank V. Paladino, George L. Shillinger, Scott R. Benson, Scott A. Eckert, Helen Bailey, Pilar Santidrián Tomillo, Steven J. Bograd, Tomoharu Eguchi, Peter H. Dutton, Jeffrey A. Seminoff, Barbara A. Block, James R. Spotila

**Affiliations:** 1 Department of Biology, University of North Carolina, Pembroke, NC 28372, USA; 2 Department of Biology, Indiana-Purdue University, Fort Wayne, IN 46805, USA; 3 Department of Natural Resources, Cornell University, Ithaca, NY 14853, USA; 4 Hopkins Marine Station, Stanford University, Pacific Grove, CA 93950, USA; 5 NOAA/NMFS/SWFSC/Protected Resources Division, Moss Landing, CA 95039, USA; 6 Wider Caribbean Sea Turtle Conservation Network, Duke University Marine Laboratory, Beaufort, NC 28516, USA; 7 Chesapeake Biological Laboratory, University of Maryland Center for Environmental Science, Solomons, MD 20688, USA; 8 Department of Biodiversity, Earth and Environmental Science, Drexel University, Philadelphia, PA 19104, USA; 9 The Leatherback Trust, Goldring-Gund Marine Biology Station, Playa Grande, Costa Rica; 10 NOAA/NMFS/SWFSC/Environmental Research Division, Pacific Grove, CA 93950, USA; 11 NOAA/NMFS/SWFSC/ Protected Resources Division, La Jolla, CA 92037, USA

**Keywords:** critically endangered species, fisheries bycatch, marine conservation, marine turtles, migratory pelagic vertebrate, satellite tracking

## Abstract

Fisheries bycatch is a critical source of mortality for rapidly declining populations of leatherback turtles, *Dermochelys coriacea*. We integrated use-intensity distributions for 135 satellite-tracked adult turtles with longline fishing effort to estimate predicted bycatch risk over space and time in the Pacific Ocean. Areas of predicted bycatch risk did not overlap for eastern and western Pacific nesting populations, warranting their consideration as distinct management units with respect to fisheries bycatch. For western Pacific nesting populations, we identified several areas of high risk in the north and central Pacific, but greatest risk was adjacent to primary nesting beaches in tropical seas of Indo-Pacific islands, largely confined to several exclusive economic zones under the jurisdiction of national authorities. For eastern Pacific nesting populations, we identified moderate risk associated with migrations to nesting beaches, but the greatest risk was in the South Pacific Gyre, a broad pelagic zone outside national waters where management is currently lacking and may prove difficult to implement. Efforts should focus on these predicted hotspots to develop more targeted management approaches to alleviate leatherback bycatch.

## 1. Introduction

Populations of leatherback turtles, *Dermochelys coriacea*, have declined precipitously in recent decades in the Pacific Ocean [1,2], resulting in their listing as critically endangered by the International Union for the Conservation of Nature. Declines result, in part, from threatening processes on nesting beaches such as beach development and the direct harvest of eggs and nesting females, but significant threats are also encountered during behaviours at sea [3,4]. Leatherbacks are the most widely distributed of sea turtles in the Pacific, and can be found in pelagic and neritic waters in tropical and temperate regions, both in the Northern and Southern Hemispheres [5]. Their broad distribution and widespread occurrence in waters of numerous countries and international commons (i.e. high seas) complicates conservation and management efforts.

For turtles at sea, incidental catch in fishing gear, or bycatch, is a considerable source of mortality [4,6,7]. Leatherbacks are captured in gillnet, trawl and longline gear in both large-scale industrial and small-scale artisanal fisheries [8,9]. Such assessments are typically made from observer and logbook data, which are sometimes voluntary and not always conducted with sufficient rigour to identify locations, timing and environmental conditions of bycatch [9]. Moreover, fisheries differ in whether bycatch data are reported publicly or recorded at all, further limiting implementation of policies to mitigate fisheries bycatch, which is particularly true on the high seas or in developing countries [10]. In the absence of detailed data on observed bycatch, targeted management to reduce or avoid bycatch requires knowledge of spatial and temporal distribution of fishing effort, an understanding of non-target species distribution and behaviour over space and time, and information on relative probability of capture should fisheries and non-target animals co-occur. Predictive models can then be developed to identify hotspots and times of potential interaction to inform bycatch mitigation strategies [7,11,12].

One of the biggest barriers to predicting bycatch events is the difficulty of collecting sufficient data in large and dynamic ocean systems, especially for species that move long distances. In the case of migratory pelagic vertebrates, detailed knowledge of ocean-scale movements has only recently come to light with technological advancements that allow individuals to be tracked via satellite for extended periods [13]. For leatherback turtles, which are highly mobile and capable of trans-oceanic migrations, several such investigations have now been completed for the two genetically distinct regional nesting populations in the East Pacific (EP) and West Pacific (WP) [14–19]. Here, we integrate information on leatherback distribution with ocean-wide data on industrial longline fishing effort to predict areas and times of potential interaction, with the aim of informing management and alleviating bycatch of this imperilled turtle. Even though bycatch in smaller-scale artisinal longline fisheries may have a significant impact on leatherback populations [20], here we focus only on large-scale industrial longlines owing to the relative availability of public data covering broad spatial and temporal scales comparable with our turtle tracking data.

## 2. Methods

### (a) Turtle movement data

Location data for adult leatherback turtles were compiled for 135 individuals tracked via the Argos satellite system from 1992 to 2008 (table 1). For the EP nesting population, deployment locations included beaches in Costa Rica (Playa Grande) and Mexico (Mexiquillo, Cauhitan and Agua Blanco), which represent the only remaining major nesting beaches on the EP coast [1]. In the WP, turtles were tracked from foraging waters off the coast of California, USA, and from nesting beaches at Jamursba-Medi and Wermon on the Bird's Head peninsula in Papua Barat, Indonesia, a location that contains 75% of all WP nesting activity [2,16]. All turtles tracked from nesting beaches were females, whereas the sample from foraging areas in the WP nesting population included both males and females. Turtles were fitted with satellite transmitters using either a towable hydrodynamic tag [14] or a harness technique [15,17,18].

**Table 1.** Summary of tracking data for leatherback turtles in the Pacific Ocean.

population	deployment location	deployment years	turtles (*n*)	mean duration (days)	min. duration (days)	max. duration (days)	mean locations per day (*n*)
eastern Pacific	Costa Rica	1992–1995	8	47	3	87	0.7
eastern Pacific	Costa Rica	2004–2007	46	300	57	568	2.3
eastern Pacific	Mexico	1993–2003^a^	26	166	9	480	2.9
western Pacific	Indonesia, California	2005–2007	55	321	22	948^b^	3.4
total (mean)			135	209			2.3

^a^Tracks do not include all years.

^b^There are some large gaps in the satellite data for this longest duration track.

Argos satellite locations were filtered and regularized at daily intervals using a Bayesian switching state-space model (SSSM) [21,22]. The SSSM couples a statistical model of the observation method (measurement equation) with a model of the movement dynamics (transition equation) [23]. Two modes are included within the transition equation providing an estimate of the animal's behaviour, indicative of migrating or area-restricted search behaviour, based on the turning angle and autocorrelation of direction and speed [19,22,24]. Briefly, the SSSM was fit using the R software package (R Developmental Core Team [25]) and WinBUGS software [26]. Two chains were run in parallel for each track for a total of 20 000 Markov Chain Monte Carlo samples. The first 15 000 were discarded, and the remaining samples were thinned, retaining every 10th sample, resulting in joint posterior distributions for each parameter based on 1000 samples. When there were long gaps in the satellite data (more than 20 days), the corresponding SSSM positions for those days were removed from the track because of high location uncertainty [19,24].

Position estimates from all years and tagging locations were compiled, and spatial use intensity was assessed for each annual quarter (quarter 1: January–March; quarter 2: April–June; quarter 3: July–September; quarter 4: October–December) at a spatial resolution of 5° × 5° using ArcGIS v. 10 (Environmental Systems Research Institute, Redlands, CA, USA). To normalize for abbreviated track lengths resulting from depleted battery power, biofouling, tag detachment or mortality, we weighted each position estimate by the inverse of the number of individuals in the sample population (EP or WP) that had position estimates for the same relative track day. The equation was specified as2.1
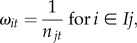
where *ω_it_* is the weight for the *t*th location estimate of the *i*th individual's track; *n_jt_* is the number of individuals of population *j* with a *t*^th^ location estimate; and *I_j_* is the set of individuals of population *j* [13]. This weighting scheme gave relatively higher value to positions from longer track durations, when fewer turtles were sampled. Thus, we imposed a threshold relative track day (85th percentile) beyond which positions received the same weight as on the threshold day. In a related study, the threshold cut-off of 85% was determined via simulation to minimize bias across various tracking scenarios [13].

To account for variation in sample sizes among quarters, we further weighted position values for each track in the population (EP or WP) according to their relative sample sizes. The population exhibiting fewer position estimates in that quarter was inflated by a factor *x*/*y*, where *x* and *y* are the number of positions for the population with higher and lower number of positions, respectively, for that quarter. The values of the weighted and normalized positions were then summed and stratified over space (5° × 5° grid cells) and time (seasonal quarters) to estimate relative use intensity. The proportional representation of summed weighted and normalized position estimates was similar among quarters (Q1 = 0.22, Q2 = 0.24, Q3 = 0.25, Q4 = 0.29). We note that transmitters were not allocated in proportion to actual nesting population size, nor did we attempt to weight data to reflect population sizes (only sample population size). Thus, our relative use intensities may not reflect actual turtle densities.

### (b) Fisheries data

Statistics on pelagic longline fisheries were compiled from the Secretariat for Pacific Communities (SPC) Oceanic Fisheries Programme and the Food and Agriculture Organization of the United Nations (UNFAO). Both organizations provide data with a spatial resolution of 5° × 5° on at least a quarterly basis and combine effort and catch statistics across a range of fisheries (see electronic supplementary material, tables S1 and S2). The primary target species for which catch statistics and effort data are compiled are tuna and billfish, though data for other species of commercial importance are also included. We accessed statistics on fishing effort (hooks) from 1990 to 2006 for longline fisheries operating in the Pacific from the SPC, and statistics on species-specific catch (tonnes) from 1990 to 2006 for longline fisheries operating in the Pacific from the UNFAO.

### (c) Turtle and fishery interactions

Our interaction models integrated spatio-temporal information on turtle use intensity, fishing effort and gear-specific capture probabilities. Longlines differ in gear configuration depending on target species, with sets targeting tuna deployed deeper (0–400 m) than those targeting billfish (0–100 m) [27,28]. Because leatherbacks spend the majority of their time in the epipelagic zone [29], they are particularly vulnerable to shallower billfish sets, though entanglement in the hooks and downlines of tuna sets also occurs [3]. For each cell and time period combination, the proportion of billfish in the total catch (per weight basis from UNFAO data; electronic supplementary material, figure S1) was used as an estimate of relative effort targeting billfish. Relative probabilities of leatherback bycatch in tuna and billfish sets were based on capture rates of 0.0246 and 0.0048 turtles per set in billfish and tuna configurations, respectively [3]. Bycatch rates were then converted to a per hook basis according to the typical number of hooks for each set type (1124 hooks per set for tuna, 850 hooks per set for billfish [28]), and a relative catchability index was calculated by dividing the number of turtles captured per hook in billfish sets by the number of turtles captured per hook in tuna sets (electronic supplementary material, table S3). For each cell and time period combination, fishing effort (hooks from the SPC data) was then multiplied by this index to adjust for gear-specific variation in bycatch probability.

To estimate relative bycatch probability, we used equations modified from Vanderlaan *et al*. [30]. First, relative density estimates were converted to relative probabilities by calculating the likelihood that a turtle occupies a grid cell *i* at quarter *t* relative to all other cells *n* across the four time periods, using the following equation:2.2
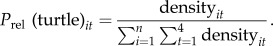


Similarly, the probability of fishing effort in grid cell *i* during the *t*th quarter relative to all other cells *n* across the four time periods is:2.3
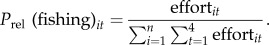


Finally, we computed an interaction index in grid cell *i* during the *t*th quarter relative to all other cells *n* across the four time periods using the equation:2.4
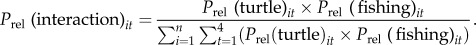
In the equations above, the probabilities for all cells in the four time periods combined sum to one, allowing for more relevant comparisons to be made among time periods.

## 3. Results

### (a) Turtle distributions

**Figure 1.**
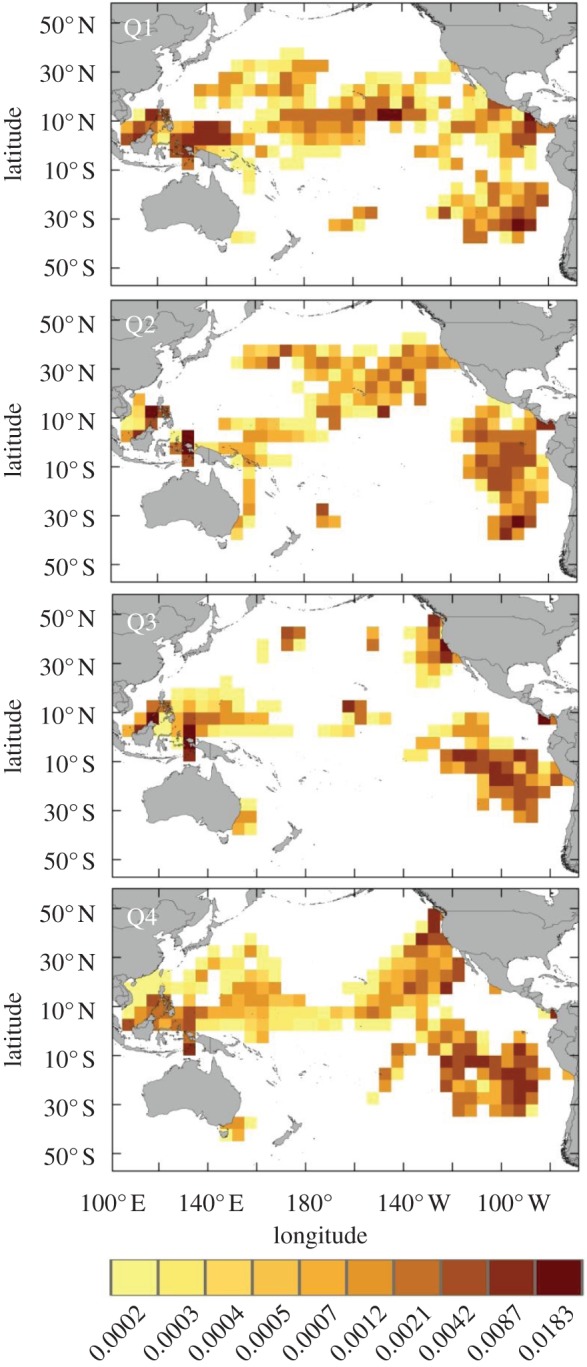
Relative use-intensity distributions for leatherback turtles in the Pacific Ocean within 5° × 5° grid cells. Values represent the proportion of all time-weighted and population-normalized positions by annual quarter such that all grid cells across the four time periods sum to one. White represents areas for which we have no data.

We generated a total of 31 074 daily position estimates for 135 turtles tracked for a mean duration of 209 days (table 1). Upper and lower 95% credible limits determined from the SSSM differed from mean position estimates by ±0.165° latitude and ±0.195° longitude. Several areas of persistent or periodic high use intensity were identified in tropical and temperate areas of the Pacific Ocean, as far west as the South China Sea, east to the Isthmus of Panama, and spanning latitudes as far as 50°N and 40°S (figure 1). However, tracks of EP and WP nesting populations did not overlap (see electronic supplementary material, figure S2*a*).

### (b) Turtle and fishery interactions

**Figure 2.**
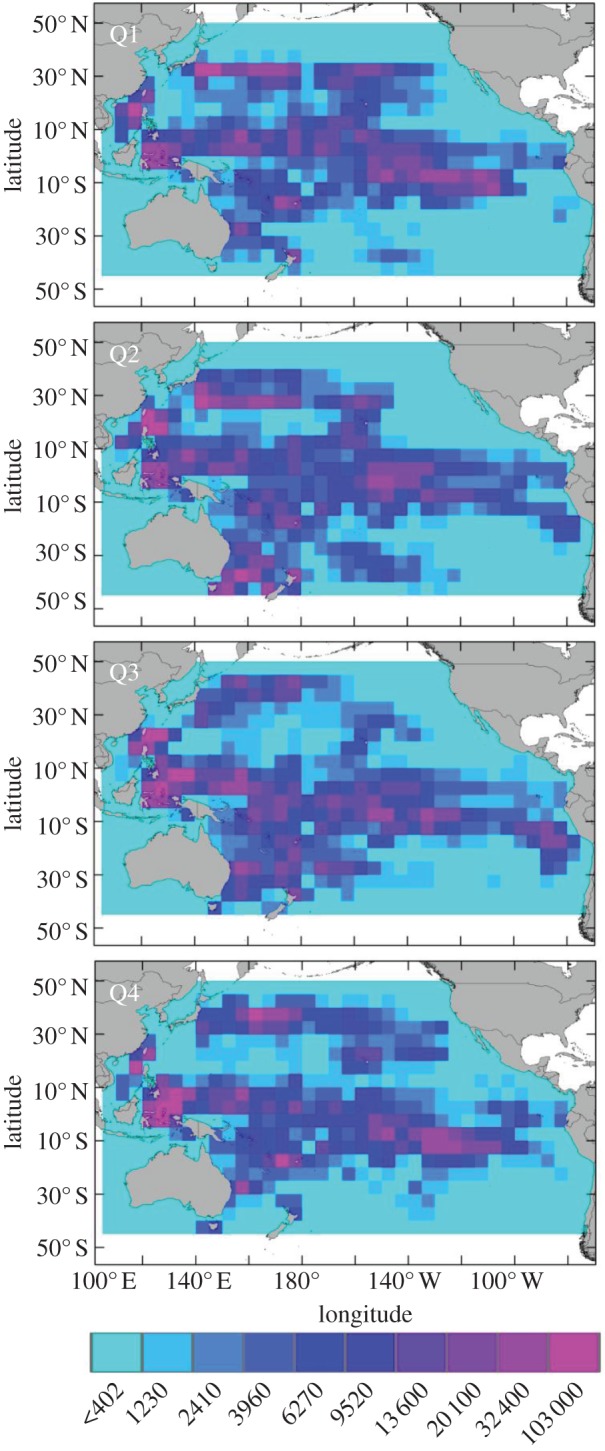
Index of longline fishing effort in the Pacific Ocean. Values are hundreds of hooks adjusted for gear-specific variation in bycatch probability within each 5° × 5° grid cell, stratified by annual quarter. White represents areas for which we have no data.

**Figure 3.**
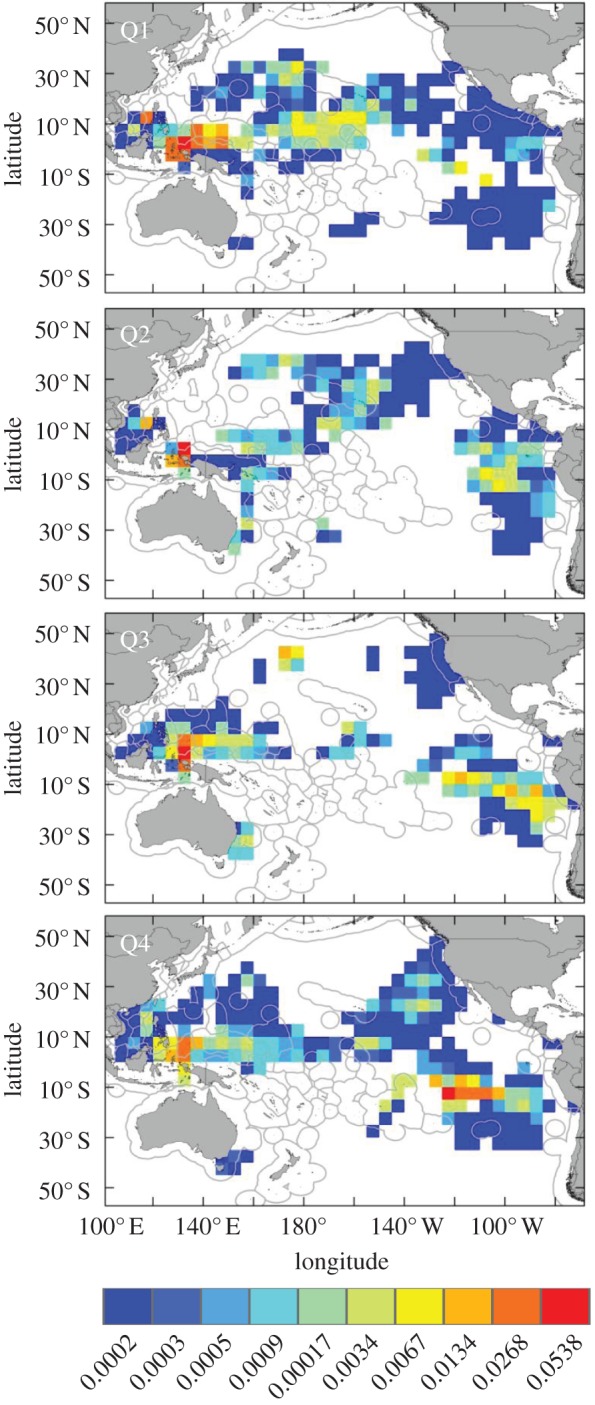
Relative interaction indices for leatherback turtles and longline fisheries in the Pacific Ocean within 5° × 5° grid cells. Values derived from the interaction index equation represent the relative proportion of bycatch risk for each time period and grid cell combination, such that all grid cells across the four time periods sum to one. Grey lines indicate national exclusive economic zones extending approximately 200 nautical miles from shore. White represents areas for which we have no data.

In the areas bounded by our satellite-tracked leatherback positions, we estimated that more than 760 million hooks have been set annually by pelagic longliners (figure 2). For turtles in the WP nesting population, we predicted consistently high bycatch risk in the tropical seas of the Indo-Pacific islands, although specific locations of interaction hotspots shifted seasonally (figure 3). Bycatch risk was predicted to be consistently greatest off the northwest coast of New Guinea, adjacent to the primary nesting beaches. This area of high interaction probability extended westward to Borneo from October through March, and eastward into the central Pacific, extending from the equator to 10–15°N, from July through March. Areas of moderate-to-high bycatch risk were also predicted in the eastern South China Sea bordering the Philippines, Palawan Island and Borneo, with peak intensity between January and June. In the central Pacific region, the bycatch risk area of broadest spatial extent was predicted to occur southwest of the Hawaiian Islands, between the equator and up to 15–20°N, from January through March. During the same season, a distinct band of moderate-to-high bycatch risk was predicted in the North Pacific Transition Zone (NPTZ) between 30°N and 35°N. From April to December, areas of predicted bycatch were more patchily distributed in the central Pacific, including immediately northeast of Hawaii. Of note are two additional predicted areas of moderate bycatch risk, one from 140°W to 120°W between Hawaii and the coast of North America from October through December, and another off the coast of southeastern Australia from April through September.

For turtles nesting in the EP, interactions were predicted to be low-to-moderate along the primary nesting migration corridor, particularly in the vicinity of the Galápagos Islands from April to June (figure 3). From April through June, areas of predicted bycatch risk shifted southwest of the Galápagos into the South Pacific Gyre (SPG), where bycatch risk was distributed over a broad spatial extent and exhibited the highest intensity between July and December. During this time, areas of moderate-to-high predicted interaction probability spanned extensively from 130°W to the coast of South America, and from the equator to approximately 30°S, peaking in intensity between 5°S and 15°S from October through December (figure 3).

## 4. Discussion

Our analysis represents the largest compilation of satellite-derived position estimates with fisheries information to predict times and locations of bycatch risk for any species of marine vertebrate. While this synthesis draws data from several independent investigations that each highlights the movements and distribution of selected Pacific leatherback population segments [14,15,17–19], the analysis of these datasets together in a standardized manner yields a rare broad-scale perspective on the overall spatial and temporal distribution of Pacific leatherbacks. Such information is essential in the design and implementation of mitigation strategies for threats that are global in distribution, such as fisheries bycatch.

Direct comparison of EP and WP nesting populations revealed that location, size and timing of predicted bycatch hotspots differed considerably, reflecting their use of different areas of the Pacific and further underscoring the need to approach these populations as separate management units [16]. For instance, we identified areas of potential bycatch risk near critical nesting locations that each present unique challenges and opportunities for bycatch management. For EP nesters, an area of potential risk occurs along the primary leatherback migration corridor between Costa Rica and the Galápagos Islands. Though this area was predicted to be of moderate bycatch risk, it occurred along a persistent migration path for nesting leatherbacks [14,17], thus representing a potential chronic threat during a critical phase in the life cycle of reproductive adult turtles. Here, turtles migrate seasonally along defined bathymetric features such as the Cocos Ridge and circumscribed within the exclusive economic zones (EEZs) of several nations with existing multinational conservation network established to manage several marine protected areas (MPAs) and world heritage sites [31]. The eastern tropical Pacific migration corridor thus presents a unique opportunity for localized management; when bycatch is constrained within EEZs, mitigation strategies may require interaction only with a limited number of fleets or vessels, facilitating implementation and enforcement of regulations in the form of gear modifications [32,33], fishery closure [34] or MPAs [35,36].

For the WP population, similar conservation networks are urgently needed, as the greatest bycatch was predicted to occur adjacent to nesting beaches in northwest New Guinea, largely confined within the EEZs of several island nations of the tropical WP (figure 3). This particular area supports the largest remaining leatherback nesting population in the entire Pacific Ocean, and considerable declines in nesting population size add urgency for development of effective management strategies [2]. However, in contrast to that of the EP nesting population, predicted bycatch risk was relatively high in all seasons adjacent to WP nesting beaches, requiring management to be appropriately protracted to reflect year-round use of the region by different boreal summer and winter nesters [2,16,18].

For animals that migrate among reproductive, foraging and wintering areas, times and areas of bycatch may vary seasonally [37,38]. When fishery and non-target species interactions vary on predictable seasonal intervals, time-area fishery closures or gear modifications can facilitate fisheries' compliance with regulations [39]. Our analyses identified several such areas where seasonal bycatch was predicted to occur as a result of temporal foraging aggregations, nesting or transiting movements by turtles. In addition to the migration corridor identified in the tropical EP region [14,17], we predicted bycatch risk to vary seasonally in seas of the tropical WP, likely reflecting seasonal movements of turtles between foraging areas and nesting beaches and temporal shifts in favourable foraging locations along shelf regions, such as in the South China Sea [18]. We also predicted seasonal bycatch risk in the ‘Café’ region between Hawaii and the coast of North America, where turtles and other predators move into pelagic waters for overwintering or while transiting to nesting beaches [13]. Areas such as the NPTZ and Tasman Sea southeast of Australia along the East Australian Current indicated possible seasonality in potential bycatch, although our ability to discern trends in these areas was limited by truncated tracks and limited sample sizes [18].

We also identified areas where potential bycatch may be spatially broad and relatively persistent, such as in the SPG, a large pelagic foraging region for EP leatherbacks [19,29]. Bycatch management becomes increasingly difficult over such broad spatial scales outside of national EEZs [40], but with modern capabilities, locations of probable interactions can be mapped in near-real time to advise bycatch reduction strategies. For instance, with remote monitoring of oceanographic conditions such as sea surface temperature, chlorophyll a concentration, currents and other variables, times and locations where prey, mobile predators and fisheries periodically aggregate can be predicted over vast areas to inform dynamic spatial and temporal zoning for fisheries management [11,41–43]. It is likely that leatherbacks move and aggregate in response to the same dynamic environmental factors that determine the density and location of their gelatinous zooplankton prey [44]. Most industrial-scale pelagic fisheries have the capacity to participate within such regulatory frameworks; they are highly mobile, can monitor environmental conditions remotely, and have the capacity to communicate rapidly with one another and with a regulatory authority [45]. An example of such a management strategy is the TurtleWatch programme in the Hawaiian-based pelagic longline fishery, where up-to-date maps of oceanographic conditions are distributed to fishery operators to advise on areas of increased likelihood of loggerhead turtle bycatch [46]. To the best our knowledge, no such predictive models have yet been implemented to inform fisheries bycatch management for leatherbacks.

An assessment of the timing and location of at-sea threats to leatherbacks on broad spatio-temporal scales is an initial critical task for the development of management strategies to mitigate threats in large and dynamic ocean basins such as the Pacific. However, this broadness of scale increases spatial and temporal uncertainty, making it more difficult to design and implement precise management strategies. The smallest spatial scale with publicly available longline data across our broad target area was of 5° × 5° resolution, requiring us to resample to the same lower spatial resolution for turtle relative density estimates, thus losing much of the finer-scale information on turtle behaviour. We did not incorporate uncertainty of turtle position estimates into bycatch predictions, but variation estimated from SSSMs was small (upper and lower 95% credible limits differed from mean position estimates by ±0.165° latitude and ±0.195° longitude) relative to the spatial scale of our modelling.

Further limitations stem from a lack of available data, requiring us to make several assumptions in our bycatch predictions. For instance, despite our large sample of turtle tracks, the staggered nature and limited duration of some tracks compelled us to assume that spatio-temporal patterns of use intensity for leatherbacks remained constant over years. Leatherbacks maintain fidelity to movement paths and foraging areas [17,18,47], though future studies examining seasonal and inter-annual variability in behaviour are necessary to further substantiate this assumption. It would be particularly important to assess leatherback spatial distribution in response to climate-driven forces such as El Niño/La Niña southern oscillation (ENSO), a phenomenon with important implications for leatherback population dynamics and ocean productivity [48]. We did not have sufficient data to rigorously examine bycatch risk in response to ENSO in all time period and population scenarios, but where data allowed for cursory comparisons, we could discern no obvious differences in turtle movements or fishing effort during El Niño, La Niña and neutral episodes (see electronic supplementary material, table S4 and figures S2*b* and S3). In addition, our data were not likely fully representative of the entire Pacific leatherback population, as deployments were largely limited to turtles leaving nesting beaches. An alternative approach would be to develop a habitat suitability model from oceanographic variables that could extend use intensity and bycatch predictions into areas and times for which we have limited or no data [11,43]. Such habitat preference models would have the additional advantage of predicting turtle responses to dynamic habitat features or periodic events (e.g. ENSO) that could then be used to inform fisheries bycatch reduction strategies [11,43,46].

Parameters with perhaps the most uncertainty in our bycatch predictions were gear-specific relative capture probabilities, which we assumed to be similar across the entire study area. In reality, capture probabilities for turtles vary among fisheries and regions [9], and perhaps over spatial scales much smaller than our 5° × 5° resolution. However, information specific to leatherback bycatch rates in different longline set types is limited for any given area, let alone from the numerous fisheries spanning the entire Pacific. We note that our estimates of gear-specific capture probabilities, with leatherbacks 6.8 times more likely to be captured in gear configurations targeting billfish relative to those targeting tuna (see electronic supplementary material, table S3), are conservative compared with those used in other studies of leatherback bycatch [7]. Also, simulations with and without adjustments for gear-specific bycatch probability resulted in largely similar predictions for the most significant bycatch hotspots, and moderate differences for areas of highest billfish effort, such as in the central and north Pacific (e.g. NPTZ; electronic supplementary material, figures S1 and S4). Clearly, numerous information gaps on both turtle behaviour and fisheries constrain the conclusions we can draw from our analyses.

## 5. Conclusion

Despite the acknowledged limitations, our analysis serves as an important approximation for use in more targeted longline bycatch management, primarily by dividing the world's largest ocean into several smaller probable hotspots where conservation efforts could now be focused. Given the broad spatial resolution of our analysis, perhaps our results could best be used to inform regional ocean planning, such as the designation of ecologically and biologically significant areas (EBSAs) [49]. The EBSA framework for marine conservation works to identify broad areas of concern that can then be targeted for further research to develop and refine management plans. Bycatch risk predicted from our models does not confirm occurrence or rate of actual bycatch, although interaction models similar to ours have performed well in predicting actual turtle bycatch timing and location [12,37]. As a next step, we advocate more regionally focused examinations of both turtle behaviour and fishery activities in high-risk areas identified here to validate predictions and tailor bycatch mitigation strategies on a context-specific basis. Although we limited our analysis to large-scale pelagic longlines, our approach could also be used to highlight areas and times of high risk in other fisheries known to capture leatherbacks, including gillnets and trawls [8,9,20], as well as artisanal and/or coastal longline fisheries. However, perhaps the most difficult current impediment to the design of effective bycatch mitigation is the obscurity of fisheries effort and bycatch data [9,10,50], especially for small-scale and artisanal fisheries that may have a disproportionally large impact on turtle populations via bycatch [20,51]. Encouragingly, several emerging partnerships between fisheries operators and biologists, aimed at sharing information, have greatly refined targeted bycatch management, and have provided examples for approaching such a complex issue as marine turtle bycatch [37,51]. Ultimately, it is in the interest of both the fishing and conservation communities to work together towards developing a clearer understanding of times, locations and conditions under which undesired bycatch of leatherback turtles occurs, to reduce these interactions, and to help alleviate the current biodiversity crisis in our oceans.

## Data accessibility

Data are deposited in OBIS-SEAMAP (http://seamap.env.duke.edu/).
